# Needs for Systems Approaches to Better Treat Individuals With Severe Asthma: Predicting Phenotypes and Responses to Treatments

**DOI:** 10.3389/fmed.2020.00098

**Published:** 2020-03-31

**Authors:** Luc Colas, Dorian Hassoun, Antoine Magnan

**Affiliations:** ^1^Nantes Université, CHU de Nantes, Plateforme Transversale d'Allergologie, Nantes, France; ^2^Nantes Université, INSERM UMR 1087, CNRS UMR 6291, Nantes, France; ^3^Nantes Université, Centre de Recherche en Transplantation et Immunologie UMR1064, INSERM, Nantes, France; ^4^Nantes Université, CHU de Nantes, Service de Pneumologie, Nantes, France

**Keywords:** asthma, type 2 inflammation, biologics, system medicine, 4P medicine, omics sciences

## Abstract

Asthma is a frequent heterogeneous multifactorial chronic disease whose severe forms remain largely uncontrolled despite the availability of many drugs and educational therapy. Several phenotypes and endotypes of severe asthma have been described over the last two decades. Typical type-2-immunity-driven asthma remains the most frequent phenotype, and several targeted therapies have been developed and are now available. On the contrary, non-type-2 immunity-driven severe asthma is less understood and still requires efficient innovative therapies. A personalized approach would allow improving asthma control with the help of robust biomarkers able to predict phenotypes/endotypes, exacerbations, response to targeted treatments and, in the future, possible curative options. Some data from large multicenter cohorts have emerged in recent years, especially in transcriptomics. These data have to be integrated and reproduced longitudinally to provide a systems approach for asthma care. In this focused review, the needs for such an approach and the available data will be reviewed as well as the next steps for achieving personalized medicine in asthma.

## Introduction

Asthma is a frequent inflammatory chronic disease affecting 240−305 million people worldwide (1), characterized by recurrent episodes of dyspnea, wheezing, chest tightness, and cough. These episodes can evolve to asthma exacerbations, defined as an acute worsening of symptoms that lead to short-acting bronchodilator agonist (SABA) and/or short-course oral corticosteroid use and/or emergency department/general practitioner (GP) unscheduled visits. Severe exacerbations can lead to intensive care unit admission and sometimes to death. Most asthma-related burdens are then due to hospitalizations, days out of work and last but not least altered quality of life (2, 3). Furthermore, asthma incidence has been increasing in industrialized countries for at least three decades, indicating a strong environmental impact (epigenetic pressure) on its pathophysiology and onset that is currently closely related to the “hygiene hypothesis and microbiota” (4, 5). Asthma therefore represents a persistent public health issue in which efforts must focus on optimizing patients' care and patients' cure for the decade to come.

Although inhaled steroids, long-acting bronchodilators such as beta-2 agonists (salbutamol, terbutaline as examples) and anticholinergics (ipratropium as an example) have positively transformed the medical support of asthma, it remains globally uncontrolled in 40–85% of patients (6) complaining of daily symptoms impairing their quality of life (both professional and personal), which leads to exacerbations. Whatever the asthma severity is, poor control remains a critical issue to handle asthma-related burdens and calls for questions: (i) Is the patient adherent to his or her treatment? If not, why? (ii) Should differential diagnosis and/or confounders be considered? (iii) Do any asthma comorbidities remain uncontrolled? (7). Should a pharmacological treatment step-up strategy or a non-pharmacological approach (allergen or occupational avoidance, smoking cessation, anxiety-controlling strategies, respiratory rehabilitation) be considered? (8). Those questions illustrate how complex asthma care is, incorporating many patient life aspects and many care practitioners. This complexity led to chronic disease management programs in which patients were supposed to actively take part in modifying their own disease and environment with the aim of reducing exacerbation rates and improving quality of life (9). This was the first attempt of personalized and participative medicine to better control the disease without modifying its natural course.

**Figure 1:**
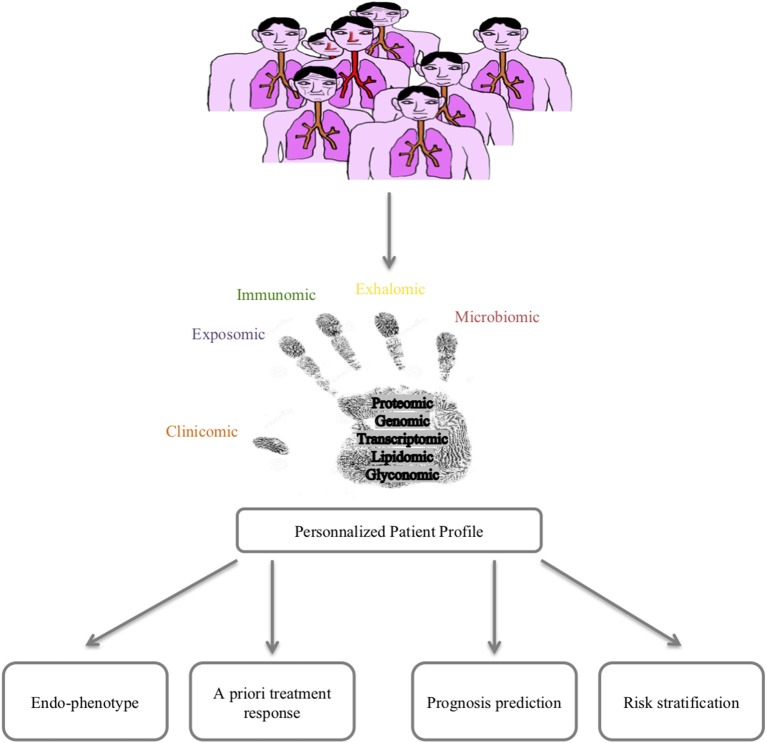
Systems medicine approach for asthma.

Lessons from the clinics are powerful tools for improving our knowledge/paradigm of a disease. Asthma is a multifactorial disease with a huge heterogeneity in clinical presentation, outcomes and response to treatment. The time has come to consider a holistic care and cure approach for asthma thanks to 4P (predictive, preventive, personalized and participative) medicine that can be best fed by new concepts and methods allowing the prediction/prevention of poor asthma control (exacerbations) and response to treatments but also the prediction/prevention of asthma onset. Moreover, such a holistic approach would allow a better understanding of asthma pathophysiology at the patient level, hence improving curative therapies. Among these 4P-related concepts, the systems approach of chronic diseases featured in asthma represents a promising route (10). The improvement in big data management obtained from omics sciences (genomic, transcriptomic, proteomic, lipidomic, glyconomic) has greatly increased the knowledge concerning asthma (Figure 1). The discussion of the state of the art of systems approaches in asthma developed below will thus concentrate on mainstream data and will not detail rapidly outdated data.

## From a Unique Disease to Multiple Phenotypes

Crosstalk between bench-to-bed and bed-to-bench studies developed during the past 2 decades has led to the emergence of new concepts. In the 1970's Asthma was described as a single disease in which airway hyperresponsiveness (AHR) was the major clinical trait and then treated with SABA. From the 1980's, a clear link between AHR and bronchial inflammation (allergic or not) was established and led to the clinical phenotypes according atopic status and/or the age of onset. To date, asthma is now considered to have a heterogenic broad spectrum of symptoms (11) with multiple phenotypes described according to clinical (age of onset, atopic comorbidities, exacerbation rate, oral corticosteroid and bronchodilator use, and quality of life as examples) and functional parameters (FEV1 and FeNO as examples) as well as multiple cellular and molecular pathways involved, making the story more complex.

### Phenotyping According to Clinical and Functional Parameters

Can clinical, functional and simple biological data be used to achieve a first step in phenotyping? To address this issue, several cohorts were set up: the Severe Asthma Research Program (SARP) in the USA (12), the Leicester cohort in the UK (13), the Airways Disease Endotyping for Personalized Therapeutics (ADEPT) cohort (14), the European Unbiased Biomarkers for the Prediction of Respiratory Disease Outcome Consortium (U-BIOPRED) cohort (15), the pooled European birth cohorts of Mechanisms of the Development of Allergy (MeDALL) (16), the Cohort for Reality and Evolution of Adult Asthma in Korea (COREA) (17), etc. Unbiased methods of clustering led to different phenotypes according to the clinical, functional and biological data chosen. Over time, the stability of those clusters was quite strong overall but varied across phenotypes (18). Although clusters might slightly differ according to the data sets used, some critical characteristics were common, including the age of onset (early vs. late onset of asthma), atopic status, obesity, comorbidities, and eosinophilic inflammation.

Early-onset asthma was mainly represented by type-2-inflammation-allergic asthma, with eosinophilic bronchial infiltration and a moderate blood eosinophil elevation (<1,000 cells/mm^3^), which was a highly stable cluster found in almost every cohort cited above. Allergen-exposure-triggered symptoms and a familial and personal history of atopic diseases (atopic dermatitis, food allergy, and/or allergic rhinitis) were often found. Data from the birth cohort (MeDALL) showed that childhood asthma, rhinitis and eczema were strongly related in symptomatic children, and this finding was interestingly dependent of IgE sensitization to pneumallergens (19). Indeed, there is a frequent association between asthma, regardless of the age of onset and its severity, and aeroallergen sensitization, ranging from 60 to 90% in house dust mite sensitization in many European cohorts (ENFUMOSA, SARP, U-BIOPRED, or TENOR) (20–22). This finding suggested a role for atopy, and, hence, type 2 inflammation, in asthma onset and/or maintenance, which was associated with an increased risk of exacerbation, especially when atopy was established in childhood. In addition, it was suggested that the serum IgE level, which is a non-exclusive biological marker of atopy, was correlated with asthma severity (23).

The role of allergens in asthmatic disease was highlighted through association studies. The most commonly incriminated allergens are house dust mites (*Dermatophagoides farinae* and *pteronyssinus*), house molds and animal dander. House dust mites are associated with a decline in FEV1 and an increase in hospitalization and exacerbation rates (24, 25). Sensitizations to domestic molds of the genera *Aspergillus* and *Alternaria* are correlated with an increase in exacerbation and hospitalization rates, and this is related to the degree of exposure (high fungal load) (26). Finally, sensitization to animal dander, especially cat dander, is associated with an increase in exacerbation and hospitalization rates in subjects who are highly exposed and with a decline in FEV1 (24, 25). In addition, recent data have evaluated the impact of house dust mite desensitization in a cohort of asthmatic patients ranging from 14 to 70 years of age with mono- or polysensitization to pneumallergens, in which there was a subpopulation of moderate to severe asthmatic individuals. The results showed in patients treated with specific immunotherapy, a significant decrease in inhaled corticosteroid doses and the exacerbation rate, all the more so when asthma was initially poorly controlled and/or moderate to severe (27). Despite these encouraging results, the disease-modifying effect of specific immunotherapy on asthma was not observed in all atopic asthmatic patients, reflecting heterogeneity in bronchial inflammatory patterns and their determinants.

Adult-onset or late-onset asthma is far more heterogeneous, with multiple clinical patterns. Nasal polyposis and aspirin-exacerbated asthma are frequent in this group, which are associated with higher severe airway obstruction and exacerbation rate (28–30).

Exacerbation-prone asthmatic individuals are mainly found among severe asthmatic patients. A recent study using the SARP data set demonstrated that exacerbation-prone asthma was significantly associated with some comorbidities [chronic sinusitis, gastroesophageal reflux disease (GERD) and higher body mass index (BMI)] but also with higher post-albuterol reversibility and blood eosinophils (31).

Obesity-associated asthma is another specific subgroup described in many cohorts. There are many clinical, functional and biological abnormalities found in obese patients to be related to asthma onset and severity (32). Considering functional data, there is a restrictive pattern with airway collapse and a higher airway hyperresponsiveness in obese patients than in non-obese patients (33). Independent of mechanical considerations, obesity also leads to significant inflammatory modulation. It can exacerbate eosinophilic inflammation in atopic asthma but also induce and worsen neutrophilic inflammation through TNFα, IL-6, and leptin pathways (34). The diagnosis of asthma in obese patients can be difficult and is often overdiagnosed.

### Phenotyping According to Granulocyte Bronchial Infiltration

**Figure 2:**
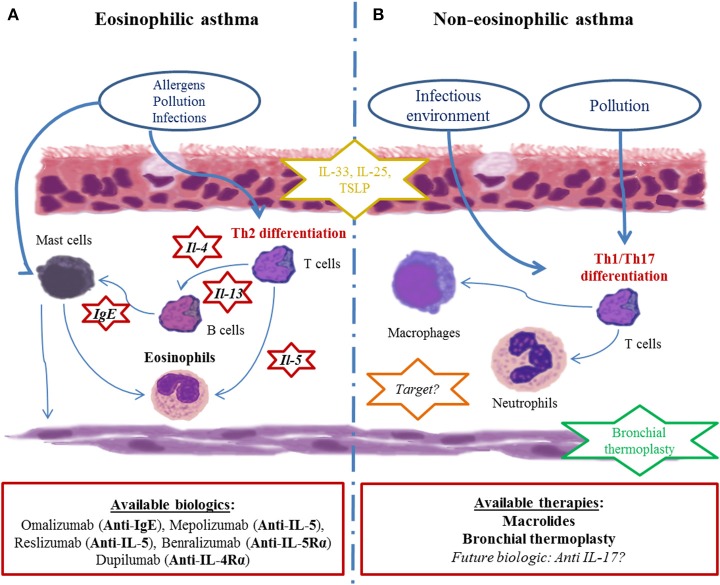
Current paradigm of asthma endotypes and related therapies. **(A)** Eosinophilic asthma is depicted as the most current clinical presentation. Driven by Th2 cytokines (IL-5, IL-4, IL-13), eosinophils chronically infiltrate bronchi and cause bronchial remodeling. Many biotherapies targeting those pathways (red-circled star) have been developed in recent decades. **(B)** Non-eosinophilic asthma physiopathology is poorly understood. Immune cells (mostly neutrophils and Th1 lymphocytes), cytokines (IL-17A and related molecules) and the immune pathway (inflammasome) seem to be involved, though their precise role remains unclear, restraining the development therapeutic drug targets (orange-circled star). Bronchial smooth muscle is an important therapeutic non-drug target because of bronchial thermoplasty (green-circled star). Subepithelial inflammatory cytokines (alarmins such as IL-33, IL-25, and TSLP) are of growing interest in both endotypes since they are far upstream of the inflammatory cascade (yellow-circled star).

In addition to clinical and functional characteristics, blood, and sputum eosinophil levels appear to be critical to define phenotypes of asthma. Since tissue eosinophilia is governed by the production of cytokines such as IL-5 and to a lesser extent by IL-13 and IL-4, it is assumed that eosinophilia either in the blood compartment or in the bronchi of asthmatic patients is a reflect of type 2 inflammation. On the other hand, pathways involved in non-eosinophilic asthma are less clear even though it is thought that non-type-2 immunity, such as type 1 and type 17 inflammation, might play an important role (35) (Figure 2). This led to the distinction between type-2- and non-type-2 phenotypes, which also arose from preclinical and clinical development of therapeutic monoclonal antibodies specifically targeting type 2 inflammation cytokines such as IL-5, IL-4, and/or IL-13 (36).

No clear threshold of blood eosinophil counts can clearly discriminate eosinophilic from non-eosinophilic asthmatic individuals. Although a blood eosinophil count higher than 1,000 cells/mm^3^ must encourage the examination of other eosinophil-related systemic diseases, blood eosinophil cell counts between 500 and 1,000 cells/mm^3^ are often correlated with eosinophilic airway inflammation. Even with blood eosinophil counts between 300 and 500 cells/mm^3^, it seems that an eosinophilic component might play a role in the pathophysiology of the disease, as shown by the relative efficacy of new biotherapies targeting eosinophils (monoclonal antibodies targeting IL-5 or IL-5Rα) in such patients (37). More recently, a transcriptomic study on airway epithelial cells either from severe asthmatic patients (smoking and non-smoking) or mild-to-moderate asthmatic patients or healthy controls could correlate for the first time a type 2 inflammation predominant gene signature (called “T2-high”) with formerly-assumed biomarkers of type 2 inflammation (blood and sputum eosinophils, FeNO, serum IgE). Blood eosinophil count >115 cells/mm^3^ were significantly though weakly correlated with a T2-high signature (38). Despite being a poor biomarker in determining asthma phenotype, high blood eosinophil counts (>1,200 cells/mm^3^) were recently associated with a higher risk of recurrent exacerbations in patients admitted to the ICU for near-fatal asthma (39).

In induced sputum, a threshold of 3% eosinophils among the recovered cells is commonly used to consider type 2 inflammation-driven asthma. Pavlidis et al., demonstrated that sputum eosinophils >1.4% were tightly correlated to type 2 predominant inflammation in severe asthmatic patients (38) rending this biomarker of great value. However, this useful technique is limited by the complicated clinical procedure that is difficult to apply in clinical practice and is only available in specialized centers (40). The rate of the expired fraction of nitric oxide (FeNO) was also proposed as a biomarker of type 2 inflammation-driven asthma (41). Indeed, FeNO roughly reflects the presence of activated eosinophils in the bronchi and is easy to measure compared to induced-sputum eosinophils. However, FeNO measurements are poorly reproducible and were shown to be poorly correlated to type 2 inflammation in asthmatic patients (38) and are rending this biomarker of limited value in daily practice. Nevertheless, it is important to identify the type 2 inflammation-driven part of the disease, since it is tightly correlated with the corticosteroid response and the risk of asthma exacerbations.

By contrast, non-eosinophilic and by extension non type 2 asthma poorly responds to corticosteroids. In biopsies from severe asthmatic patients, Wenzel et al. previously reported the existence of neutrophilic and pauci-granulocytic patients (36). An analysis of induced sputum can also distinguish these two patterns of non-eosinophilic patients, although no clear threshold is observed for neutrophils. The same observation was made for the neutrophil blood count. Non-type-2 (neutrophilic/pauci-granulocytic) asthma would therefore appear as an orphan disease, with some drugs such as macrolides being unequally efficient (42), and new drugs being far upstream of any evidence-based interest (anti-IL-17, and drugs targeting epithelial-derived cytokines such as TSLP, IL-33 or IL-25) (43). Recently, Östling et al., on the behalf of U-BIOPRED study group, identified an IL-17-high endotype in the bronchial epithelial cell transcriptome of severe asthmatic patients. The gene signature associated with that endotype was highly similar to the one observed in psoriasis, suggesting anti-IL-17 therapies as a treatment option for those patients (44).

## From Phenotypes to Targeted Therapies: Toward Deciphering Multiples Mechanisms

From that point of view, asthma could be divided into two main endotypes: type 2 and non-type-2. The former is more frequent, corticosteroid responsive and targeted by several new biologics, whereas the latter is more rare, difficult to treat and comprises obese patients (45). In many patients, it should be considered that type 2 and non-type-2 inflammation are not mutually exclusive. In so-called type 2 asthmatic individuals, a contingent of non-Th2 lymphocytes and neutrophils co-infiltrates the bronchi together with Th2 lymphocytes and eosinophils (46). Such mixed phenotypes make the story more complex in understanding asthma heterogeneity, though they should be considered in asthma care. Thus, cluster analysis of clinical, functional and basic inflammatory data from large cohorts has permitted the identification of several phenotypes of asthma during the last two decades. These phenotypes are more or less overlapping and are sometimes associated in the same individuals, making it difficult to set up strategies of phenotype-based personalized care (Figure 2).

To understand the pathophysiological pathways involved in the different asthma phenotypes previously described, several molecular studies were run using a modern systems biology unbiased approach with the underlying assumption that discovering associated endotypes would lead to new therapeutic targets.

Woodruff et al. were among the first to identify a panel of 22 differentially expressed genes (mRNA array analysis) in bronchial epithelial cells from mild allergic asthmatic and non-asthmatic patients on the basis of corticosteroid-responsive and tobacco-smoke-induced genes (47, 48). The underlying hypothesis of this novel work was based on epithelial dysfunction driving type 2 inflammation in bronchi due to allergen exposure. These 22 differentially expressed genes could thus reflect new phenotypes and endotypes and new therapeutic targets. Then, the researchers focused on three genes, POSTN (periostin), CLCA1 (chloride channel accessory 1), and SERPINB2 (serpin family B2), which were identified as being upregulated by IL-13 and inhibited by steroids, in a type-2-immunity-driven strategy. Later, high levels of serum periostin proved to be a reliable and promising companion biomaker for identifying a subgroup of severe type-2-immunity-driven asthma patients responding to anti-IL-13 biologics (Figure 2). Though, one of the main limit was the weak number of asthmatic patients under inhaled corticosteroid (ICS). Unfortunately, anti-IL-13 biologics were not as active as expected in severe asthmatic patients to allow further pharmaceutical development (49). It is important to bear in mind that the analysis of serum periostin and anti-IL13 biologics demonstrated that (1) the so-called type-2-immunity-driven phenotype is heterogeneous with an unequal importance of each cytokine pathway in each patient; (2) it is unlikely that a single companion biomarker can provide a specific signature for a specific endotype/phenotype/target; (3) although attractive as a serum assay, serum periostin levels have not proved to be a better discriminant of the type-2-immunity-driven phenotype than blood and sputum eosinophil counts and FeNO, especially when it comes to severe asthma (38, 50); and (4) there is a need for companion markers that are able to predict the response to targeted therapies.

Although conventional drugs, both inhaled and systemic steroids and bronchodilators, are designed to be efficient in any asthmatic patient with significant side effects, new drugs have arisen from the better characterization of the underlying mechanisms mostly in type-2-immunity-driven asthma in the last 20 years. These new drugs are monoclonal biologics targeting specific cytokines or their receptors (IL-5, IL-4Rα, IL-5Rα) and are expected to be efficient in selected patients only. The main limits to date of these novel specific immunomodulatory drugs are their high production cost and the lack of reliable companion biomarkers that allow the prediction of the individual response a priori.

Omalizumab is an anti-IgE monoclonal antibody that appeared on the market more than 10 years ago and was approved for severe asthmatic patients with proven allergic involvement. The efficacy of omalizumab should be assessed after 16 weeks in terms of the improvement of asthma control and the reduction of exacerbation rate. Several *post-hoc* studies showed that higher baseline clinical type 2 immunity biomarkers such as blood and sputum eosinophils and FeNO were positively correlated with anti-IgE monoclonal antibodies responsiveness. Interestingly, no correlation were found between serum IgE and atopic/allergic status (38, 51–54) (Figure 2). However, ~25% of severe asthmatic patients remained unresponsive to anti-IgE monoclonal antibodies despite presenting with higher baseline type 2 immunity biomarkers. This suggests that more accurate biomarkers or combinations of markers are needed.

Mepolizumab and reslizumab are anti-IL-5 monoclonal antibodies, albeit benralizumab is an anti-IL-5Rα monoclonal antibody, all of which specifically target eosinophils. They have been approved in severe eosinophilic asthma. For these drugs, blood eosinophil count is proposed as a predictive biomarker: the more elevated the blood eosinophil is, the more efficient these drugs are (55). Nevertheless, a proportion of patients, which is yet to be determined in real-life studies, will not respond to these biologics, even with a consequent eosinophil count reduction. Again, this suggests that more accurate biomarkers or combinations of markers are needed (Figure 2).

Other biologics targeting type 2 immunity, such as anti-IL-4Rα (targeting both IL-4 and IL-13, dupilumab) (56) or anti-TSLP (tezepelumab) (57), are to date in phase II or III trials in type-2- and non-type-2-immunity-driven asthma (Figure 2). They are also commercialized in other non-allergic type-2-immunity-driven diseases such as atopic dermatitis (58). These novel drugs are not restricted to eosinophilic asthma or eosinophil-mediated disease. Additionally, IL-33, a cytokine for which a major role was demonstrated in fungus- and virus-induced asthma exacerbations in mouse models but also in moderate asthmatic patients (59, 60), is a target under investigation.

At that stage, it clearly appears that asthma represents a highly heterogeneous entity with many clinical, functional and biological phenotypes intricately involved. Asthma is therefore a chronic disease for which precision medicine must be implemented, especially since targeted expensive drugs are arising. To this aim, reducing asthma to 2 entities, Type 2 inflammation-driven asthma and non-type 2 inflammation-driven asthma, is not satisfactory, and more is needed to further refine asthma phenotypes.

**Figure 3:**
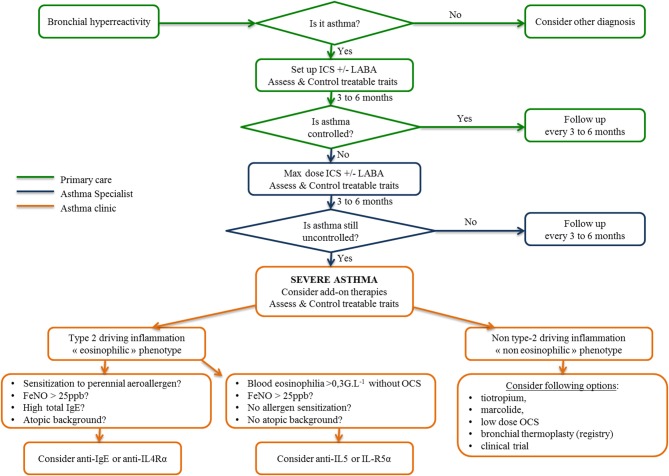
Algorithm for asthma management from primary care to the asthma clinic inspired by the 2018 GINA recommendations, “From difficult to treat to severe asthma.” ICS, inhaled corticosteroids; LABA, long-acting beta agonist; OCS, oral corticosteroids.

Facing the increasing development of biologics in asthma treatment, international consensus for asthma management has restricted the use of biologics to severe refractory asthma, whose definition was internationally defined recently (5, 61). Today, the diagnosis and management of asthma is based on defining a personalized and label-free phenotype with *ad hoc* drug and non-drug therapies. To this purpose, Agusti et al. proposed the term “treatable traits,” defined as clinical, functional, biological and psychosocial characteristics classified into three categories (pulmonary, extrapulmonary, and psychosocial), for which therapeutic interventions (drug and non-drug therapies) are possible in theory and linked to an improvement of chronic respiratory disease (62), as depicted in Figure 3. First, patients displaying respiratory symptoms concordant with asthma should undergo a thorough clinical examination and pulmonary function tests to exclude all other diagnoses and document bronchial reversibility and/or hyperresponsiveness. When asthma is confirmed, according to up-to-date guidelines, inhaled corticosteroids eventually combined with another controller will be proposed. Treatable traits focusing on extrapulmonary and psychosocial aspects have to be checked and addressed at that stage (primary care). If asthma remains poorly controlled (difficult-to-treat asthma), asthma specialists will be involved to optimize inhaled treatment, reassess differential diagnosis and check and address more treatable traits, especially pulmonary traits (secondary care) (7). When these steps are achieved, if asthma is still uncontrolled, a biologic can be considered as an alternative or add on to a low dose of daily steroids. This requires to determine which type of inflammation is driving the phenotype of these severe asthmatic individuals, according to clinical, functional and biological data as previously described (61, 63). In type 2 eosinophilic allergic patients, anti-IgE or anti-IL4Rα should be considered first, whereas in type 2 eosinophilic non-allergic asthmatic patients, anti-IL5 and anti-IL5-Rα should be proposed first. To date, no biologic or drug specifically targeting non-type-2 inflammation is available probably due to (1) the relative scarcity of that phenotype compared to type-2 asthma; (2) the ignorance about its physiopathology making it complex to identify therapeutic targets. Low-dose OCS frequently remains the sole option despite long term side effects (diabetes mellitus, increased atherosclerosis, dyslipidemia, high blood pressure, weight gain, osteoporosis, adrenal insufficiency, muscular atrophy, higher frequency of chronic glaucoma and cataract) and variable efficacy in such non-type-2 cases to decrease annual exacerbation rate. OCS related co-morbidities comprise a huge part of the economic and also medical burden in severe asthma. Indeed in a recent study, Barry and al. estimated that non-asthma medication comprised on average from 25 to 30% of the annual cost in severe asthmatic patients (64). It reached up to 80% for diabetes mellitus or osteoporosis as showed in the PACEHR study (65). As a consequence, severe asthmatic patients are more susceptible to be disabled and/or die from OCS related co-morbidites than fatal asthma attacks. As a conclusion, OCS chronic prescription in severe asthmatic patients should be initiated in asthma clinic. The benefit/risk ratio should be followed up at regular intervals or stopped all the more so patients poorly respond. In exacerbation-prone non-eosinophilic asthma, the long-term use of macrolides or bronchial thermoplasty (66) can be discussed. Finally, a clinical trial must be proposed for patients remaining symptomatic despite all available therapies.

## Toward a Systems Approach in Asthma: Unbiased Omics Studies (Figure 4)

Wheelock et al. nicely reviewed sophisticated large-scale analytical methods to quantify gene expression (transcriptomics), proteins (proteomics), lipids (lipidomics), and metabolites (metabolomics) in various samples, including blood, and as far as pulmonary diseases are concerned, induced sputum, bronchial biopsies, epithelial brushings, and bronchoalveolar lavages (67). Moreover, measurements of volatile organic compounds (VOCs) found in exhaled air and exhaled air condensates (volatolomics, exhalomics) can be addressed as well as the integration of inhaled pollutants and other allergens (exposomics), and more recently characterized pulmonary microbiota (68–70) (Figure 1).

**Figure 4:**
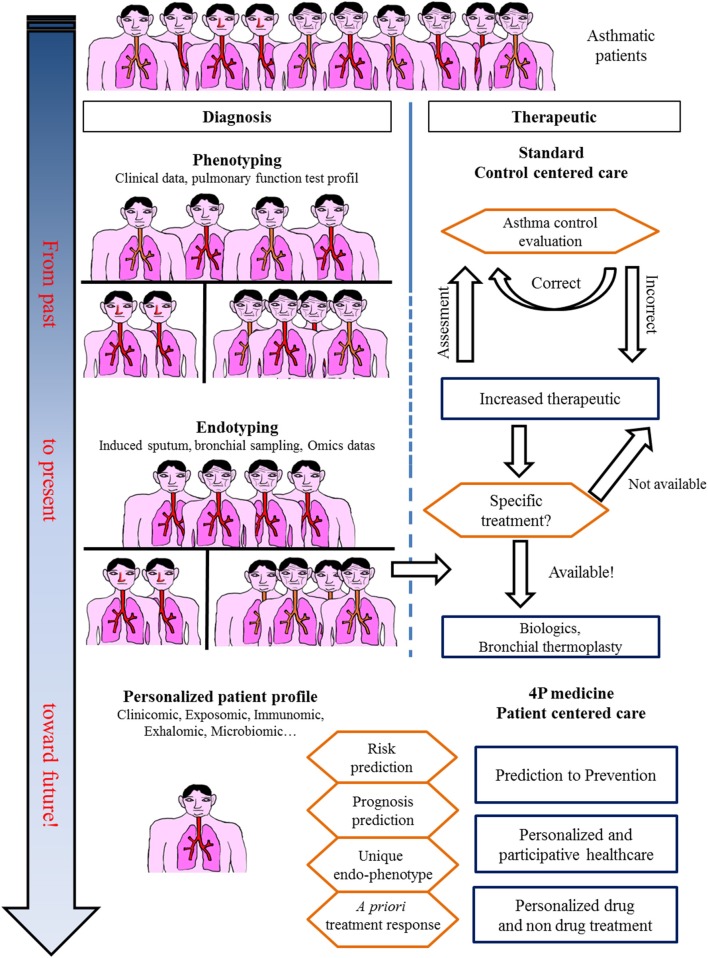
In the past, asthma was considered a single disease. Treatment was centered only on obtaining control. Today, asthma care, especially for severe asthmatic patients, takes into account phenotypes and endotypes in treatment decisions. The future for asthma care is an integrative approach with a personalized profile leading to specific care.

### From Phenotypes to Endotypes Using Unbiased Transcriptomic Approach

To date, omics data in asthma mostly concern mRNA analysis in various compartments (blood, epithelial cells, induced sputum, and bronchial alveolar lavages) obtained from large multicentric cohorts. In the SARP study, bronchial epithelial cell mRNA expression was analyzed in relation to FeNO in 155 individuals with severe asthma and in healthy volunteers (71). On the basis of 549 genes that correlated with FeNO and clinical/functional characteristics, 5 clusters were distinguished, differentiated by 1,384 genes. These genes in turn segregated into 9 gene clusters. Networks of genes related to the type 2 immunity pathway and unknown pathways in asthma pathophysiology were identified. A first Australian report identified upon sputum expression profiling three groups of patients roughly corresponding to what was known from previous works on cellular sputum profiles, i.e., eosinophilic inflammation (group 1), neutrophilic inflammation (group 2), and a pauci-inflammatory profile (group 3) (72). Because of an unbiased approach, this work identified a series of genes related to each phenotype. In a second paper, the same authors validated a six gene pattern that could discriminate eosinophilic from neutrophilic and paucigranulocytic asthma (73). They further explored the extrinsic and intrinsic parameters of this set of genes (i.e., sensitivity, specificity, positive, and negative predictive values) both with blood and sputum eosinophils to predict OCS response (74). The genes were thus chosen from the results of previous studies, with 3 being associated with eosinophilic asthma (CLC, CPA3, and DNASE1L3) and the 3 others being more related to neutrophilic asthma (IL-1β, ALPL, CXCR2). They found one gene (CPA3) to be upregulated before corticosteroid treatment in responder patients, 2 genes for which expression decreased after treatment (CPA3 and CLC), and the 4 other genes remained stable. Importantly, the receiver operating characteristic (ROC) curve showed a better performance of the six gene patterns in predicting OCS response than that of either blood or sputum eosinophils.

A vast amount of transcriptomic data in asthma was published within the last 2 years in the framework of the U-BIOPRED (Unbiased Biomarkers in Prediction of Respiratory Disease Outcomes) European study. In peripheral blood, a severe asthma signature was detected, both in a training and a validation cohort of severe asthmatic subjects compared to non-asthmatic subjects (75). A total of 1,693 differentially expressed genes were identified. Adjustment to the number of circulating cells, which is different between asthmatic and non-asthmatic subjects, reduced the signature to 268 genes. To assess whether the results were OCS dependent (40% of the severe asthmatic subjects included), the analysis repeated in non-OCS-treated patients showed that 30% of the severe asthma signature was independent of OCS. Hierarchical clustering separated severe asthmatic subjects into 2 clusters, one containing 87% of patients with severe asthma and most of the OCS-treated subjects and the other containing 58% of patients with severe asthma and 85% of non-asthmatic patients and mild/moderate asthmatic patients. A series of genes were downregulated in severe asthmatic patients, including B cell-related genes, especially in OCS-treated patients, as expected. T lymphocyte-related genes were in turn of higher expression. The choice of blood compartment to detect signatures specific to bronchial disease is questionable. However, it is widely recognized that blood eosinophils and even serum allergen-specific IgE are suitable biomarkers to consider the eosinophilic or allergic status of asthma and the possibility of using anti-IL5 or anti-IgE monoclonal antibodies, respectively (Figure 3). In addition, as the systems approach is set up to detect predictive biomarkers suitable for daily practice in the clinic, the biomarkers should be non-invasive, reproducible and easy to collect. Finally, asthma can also be considered as part of a systemic disorder, especially when allergic asthma is considered.

Nevertheless, several U-BIOPRED studies reported on transcriptomics from bronchial samples (induced sputum and epithelial cells). Lefaudeux et al. studied the transcriptome and proteome in induced sputum in a training cohort of 266 asthmatic patients and in a validation cohort of 152 patients (76). Four clusters were identified in both cohorts, among which 3 clearly included the most severe patients. Two clusters were related to chronic airflow obstruction, with one associating smokers and ex-smokers with late-onset asthma and the highest blood eosinophil counts. A third cluster contained non-smokers with OCS therapy and a fourth cluster contained obese females with recurrent asthma exacerbations and normal lung function. Importantly, these clusters are reminiscent of those from previous cluster studies performed in the SARP and Leicester cohorts (12, 13).

Another study of sputum transcriptomics from U-BIOPRED identified 42 genes upregulated in patients with severe asthma compared to mild to moderate asthmatic patients (77). This signature included the IL1 receptor family and inflammasome-associated genes. These genes were also differentially expressed according to the granulocytic status of asthmatic patients (eosinophilic vs. neutrophilic), where IL-13 gene upregulation was associated with eosinophilic asthma. Interestingly, the K Baines group in Australia recently detected inflammasome protein gene expression and notably NLRP3 to be increased in asthmatic sputum and singularly, but not only, in obese asthmatic patients (78). An additional U-BIOPRED paper by Kuo et al. compared mRNA expression in induced sputum reported in 3 transcriptome associated clusters, so-called TACs, with one TAC being characterized by highly type-2-immunity-driven asthma with eosinophilia in the tissue and blood and 2 other non-type-2-immunity-driven TACs characterized by metabolic/mitochondrial and again inflammasome-associated pathways (79). These observations are consistent with two recent studies in which the epithelial IL-6/inflammasome trans-signaling pathway was associated with a severe asthma phenotype prone to a high exacerbation rate, neutrophilic inflammation in sputum and a high BMI index independent of atopic status (80, 81). More precisely, IL-6 trans-signaling was shown to amplify the local inflammatory response and epithelial dysfunction in the lungs, suggesting this mechanism as a potential therapeutic target (81).

Transcriptomics were also applied in induced sputum but also in nasal and bronchial brushings to determine the differential expression of genes in these different compartments within the lungs of asthmatic patients since epithelial dysfunction at asthma onset is widely acknowledged today (82). In this paper, severe adult-onset asthma patients were compared to childhood-onset patients. Differentiating signatures were found in all three compartments, and interestingly, the assignment of gene networks to specific pathophysiological pathways allowed the differentiation of several distinct signatures within each compartment. Specifically, genes related to eosinophilic inflammation, ILC3 and mast cells were upregulated in late-onset asthma. From the same authors, the same study design was used to distinguish patients with persistent airway obstruction. This time, signatures related to the increase in eosinophilic inflammation and IL-13 and to the decrease in IFN-γ were identified in persistent obstructive patients, together with genes associated with lung injury and remodeling (50). An additional paper on epithelial brushings and bronchial biopsies determined 4 clusters of patients: 2 were characterized by high expression of type 2 immunity cytokines and a lack of corticosteroid response and differed in the tissue distribution of eosinophils. Another cluster included patients with a higher body mass index. The two other groups were mainly non-eosinophilic (83).

### From Phenotypes to Endotypes Using Unbiased Proteomic/Metabolomic Approach

Beyond transcriptomic analysis, proteomics and metabolomics will also provide new interesting data. For this purpose, the use of exhaled condensates and exhaled air would provide non-invasively obtained lung-derived samples of VOCs (proteins and metabolites) (84). Such samples can easily be taken several times in the same patient and therefore provide longitudinal data that can be integrated with mathematical models, providing for each patient an individual trajectory that can be compared to that of all other patients of a longitudinal cohort (85). Probabilities for exacerbations and response to treatments would then be addressed. Some data have already emerged. Brinkman et al. from the Amsterdam group compared the VOCs at baseline with those of exacerbation and recovery in patients in whom asthma treatments were withdrawn on purpose. eNose correctly classified patients in 21/22 cases of exacerbation compared to baseline and in 17/22 patients of exacerbation compared to recovery (70). The same authors also demonstrated, in an unsupervised approach using exhalomic analysis over an 18-month follow-up, 3 stable clusters of severe asthmatic patients. Two of the clusters referred to the main type of immunity implied (eosinophilic vs. neutrophilic), whereas the other cluster referred to OCS use (69).

A recent unbiased proteomic study on induced sputum supernatant from 200 asthmatic patients (moderate and severe asthma) and 46 healthy volunteers identified 10 subendotypes comprising 247 differential proteins. Eight out of 10 could be meta-classified as eosinophilic (3 subendotypes), neutrophilic (3 subendotypes), or paucigranulocytic but highly atopic (2 subendotypes) according to sputum myeloid cellularity, and the clinical characteristics and transcriptomic pathways were consistent with the 3 TACs described by Kuo et al. (86). The authors also identified predictive biomarkers associated with the 3 main proteotypes. The eosinophilic proteotype was associated with the upregulation of pro-type-2 immunity proteins mostly via an inhibition of neutrophil activation (transthyretin, serotransferrin, alpha1 anti-trypsin) or via the activation of eosinophils (IgG3, C3, histone H4) (86–88). Conversely, the neutrophilic proteotype was associated with the upregulation of proteins secreted by neutrophils (azurocidin, S100, annexin A, neutrophil gelatinase-associated lipocalin, myeloperoxidase) (80). Interestingly, in a hypothesis-based approach, Barbaro et al. detected the exhaled matrix metalloprotease-9 (MMP9) in different phenotypes of asthma and found it elevated in severe neutrophilic asthma (89), echoing the results of both the aforementioned study by Schofield et al., where MMP9 was associated with neutrophilic inflammation in bronchial mucosa from asthmatic patients (86), and those of a previous study in the field of lung transplantation that showed that the serum MMP9 level was predictive of bronchiolitis obliterans syndrome in lung transplant recipients, a condition also related to airway neutrophilia and bronchial remodeling (90).

### From Phenotypes to Endotypes Using Unbiased Microbiomic and Exposomic Approach

Coupled with biological data coming from tissue- or blood-derived analysis, environmental data and functional microbiomic will provide new insights in underlying mechanisms allowing a better precision medicine approach at individual level. Large databases already exist that are capable of assigning to each individual his or her cumulated exposure to various environmental pollutants. Longitudinal patient data can therefore be coupled to these databases and easily reveal relationships between lung function and pollutant exposure as an example (91). Asthma predisposition factors in early life (parental smoking, RSV and RV infection, asthma in relatives, early sensitization to aeroallergen, atopic dermatitis) are composed of inherited and environmental factors (92–94) where the later seems to play an important role via the hygiene hypothesis and more precisely via microbiota as showed by large epidemiological studies (5, 95, 96). Concerning microbiota, a recent Australian study examined the microbial composition of induced sputum from asthmatic patients and showed that neutrophilic asthma was associated with a different airway microbiology from that seen in other inflammatory phenotypes (97). Further mechanistic studies are needed to better understand the reciprocal interactions and role of the airway microbiota, environmental exposure to pollutants and early life predisposition factors in asthma physiopathology.

## Future Directions: From Omics to “Treatable Mechanisms”

Omics sciences in asthma have started generating a vast amount of data allowing us (1) to better decipher physiopathological pathways and triggers, (2) to better characterize endotypes/phenotypes (clusters of patients), and (3) to better predict exacerbations and/or response to treatments. However, omics data today remain of unequal quality and are unstandardized, rendering comparison and/or extrapolation for modeling the diagnosis or prognosis of asthma difficult. A large effort toward omic data standardization must be considered before defining what the next steps will be, as proposed by the TRIPOD statement (98). Indeed, every unbiased study previously cited naturally attempts to correlate omics-identified patterns (endotype) to well-known phenotypes or characteristics of asthma (eosinophilic vs. non-eosinophilic, severe vs. non-severe, persistent obstructive vs. others, obese vs. non-obese), adding more accurate knowledge to the pathophysiology of asthma and proposing candidate biomarkers for diagnosis and/or prevention. Another perspective from omics sciences is the rising concept of “treatable mechanisms” to replace phenotypes. Indeed, omics sciences have highlighted numerous up- or downregulated pathways that represent potential robust biomarkers bearing enough precision necessary for 4P medicine to better model asthma and provide “care and cure” at the level of a single patient and thus move from dissociated fingerprints to integrative handprints (Figures 1, 4) (99, 100). Though, one must bear in mind that omic (i.e., molecular phenotype) approach provide association between phenotype and molecular pathways. Mechanistic studies are needed to better decipher/define ≪treatable mechanisms≫ and then better define endotypes and therapeutic targets.

Another important question is “How stable are asthma clusters/endotypes/phenotypes and their companion biomarkers in the life span of an asthmatic patient?” A retrospective study based on clinical and functional parameters in 1,325 asthmatic patients with a 20-year follow-up identified 7 baseline clusters of patients. An interesting point is that only 1/5 of the patients moved from a cluster to another during follow-up (18). This question has been addressed by preliminary unbiased studies using omics sciences, such as in the ADEPT cohort, in which a stable and reproducible clustering of patients was found through external validation in the U-BIOPRED cohort over a 12-month follow-up (101). However, these encouraging results need to be confirmed using longitudinal data to achieve an efficient systems approach for modeling asthma “care and cure” in a precision medicine outcome.

In conclusion, recent years have provided a huge amount of data on the heterogeneity of asthma, and the large multicenter initiatives taken are of inestimable value. These data do not provide biomarkers that are ready to use in clinical practice; a great deal of clarification work must be initiated. In addition, longitudinal cohorts aggregating standardized data of various nature for each patient are necessary to achieve a systems-based approach precision medicine for asthma care and cure (Figures 1, 4).

## Author Contributions

AM provided the main concepts of the manuscript (ideas, stratifications, and bibliography). AM, DH, and LC drafted the manuscript. DH and LC conceived the figures and legends and critically revised the manuscript. All of the authors approved the final version of the manuscript.

### Conflict of Interest

During the last 5 years, AM has received non-financial support from GSK, Novartis, Boehringer, Astra Zeneca, and Chiesi. The remaining authors declare that the research was conducted in the absence of any commercial or financial relationships that could be construed as a potential conflict of interest.
